# Long-term follow-up of inpatients with traumatic fractures who received integrative Korean Medicine treatment: A retrospective analysis and questionnaire survey study

**DOI:** 10.1097/MD.0000000000034530

**Published:** 2023-10-13

**Authors:** Min Kyung Kim, Kyoung Sun Park, Gyu Cheol Choi, Jae Eun Yu, Hee Won Lee, Yong Su Kwon, Hyo Seung Huh, Suna Kim, Eun-San Kim, Jinho Lee, In-Hyuk Ha, Yoon Jae Lee

**Affiliations:** a Daejeon Jaseng Hospital of Korean Medicine, Daejeon, Korea; b Jaseng Hospital of Korean Medicine, Seoul, Korea; c Jaseng Spine and Joint Research Institute, Jaseng Medical Foundations, Seoul, Korea.

**Keywords:** disability index, EQ-5D-5L score, Korean medicine, traumatic fractures

## Abstract

Previous studies have reported pain reduction after Korean medicine (KM) treatment in patients with fractures. However, these studies were limited by small sample sizes and short observation periods. To address these limitations, we aimed to analyze the outcomes of patients with traumatic fractures who received integrative KM treatment and investigate their long-term progress through follow-up observations. This study was a retrospective analysis and questionnaire survey conducted at a multi-center inpatient care setting in Korea. A total of 1150 patients who had traumatic fractures and received at least 5-day inpatient care at one of 5 KM hospitals. Finally, 339 patients completed the follow-up survey. The questionnaire survey was administered 3 months post discharge. The primary outcome was the difference in numeric rating scale (NRS) scores at admission and discharge for fracture-related pain. The secondary outcomes were EuroQol 5-Dimension 5-Level (EQ-5D-5L) score, Oswestry Disability Index, Neck Disability Index, Western Ontario and McMaster Universities Arthritis Index, Shoulder Pain and Disability Index, and Patient Global Impression of Change (PGIC) score. The follow-up questionnaire survey included questions on surgery and imaging before admission and after discharge and treatment within the past 3 months. The mean NRS score at follow-up showed a significant decrease of 4.41 points compared with that at admission (*P* < .001). The mean EQ-5D-5L score at follow-up showed a significant increase of 0.18 points compared with that at admission (*P* < .05). In the follow-up survey on PGIC, 307 participants (90.56%) were “minimally improved” or better. Integrative KM treatment can help improve pain, functional impairment, and long-term quality of life in patients with traumatic fractures.

## 1. Introduction

A fracture, which is a loss in the structural continuity of a bone or cartilage due to the excessive application of force, may be caused by an external force, such as trauma, or may be a result of a pathological condition that weakens bones, such as osteoporosis.^[1,2]^ According to data provided by the Korean Health Insurance Review and Assessment Service, the number of patients treated for fractures each year is increasing. Contributing to this trend are the aging population and an increased number of traffic accident victims.^[3]^ The number of patients undergoing treatment for fractures has increased consistently from 2,255,134 (4.4% prevalence) in 2016 to 2,503,502 (4.84% prevalence) in 2019. Moreover, fracture-related medical costs increased from 1,556,661,414 Won in 2016 to 2,093,066,351 Won in 2019.^[4]^

In addition to the direct medical costs associated with fracture treatment, fractures can directly or indirectly cause other long-term problems. First, a fracture can lead to an additional fracture in another area or increase the risk of other chronic comorbidities. Moreover, fractures can lead to functional impairment and mobility loss, which are highly associated with depression, reduced quality of life (QoL), and loss of productivity.^[5]^

Fracture treatments, namely, conservative treatment and surgery, are determined based on the fracture pattern. Surgical intervention, including internal or external fixation and open reduction, is primarily applied in complex fractures such as open, comminuted, or multiple fractures. For non-complex fractures, conservative treatment may be applied, including closed reduction, fixation (including splints, casts, and traction), analgesics (paracetamol, opioids, and nonsteroidal anti-inflammatory drugs), and nerve blocks.^[1,6]^ In Korea, which has a dual healthcare system, non-surgical patients or patients undergoing postoperative bone union and rehabilitation may choose Korean medicine (KM) treatment as conservative treatment. KM is based on traditional treatment approaches; it combines Korea’s independent sa-sang theory with traditional Chinese medicine and primarily makes use of acupuncture, herbal medicine, and chuna manipulation.^[7]^

A retrospective observational study showed pain reduction after KM treatment in 17 patients with hip fractures.^[8]^ Another randomized controlled trial (RCT) revealed pain reduction and function recovery following acupuncture in 97 patients with intertrochanteric fractures.^[9]^ A systematic review on patients with humeral fractures showed significantly lower levels of pain in the acupuncture group than in the control group.^[10]^ However, several studies have included only small sample sizes, and case studies account for most of the studies to date. Meanwhile, follow-up observational studies on the long-term effects in patients with fractures are still lacking. Accordingly, the aim of this study was to analyze the outcomes of patients who received integrative KM treatment for traumatic fractures and examine the long-term progress of the therapeutic effects of integrative KM treatment.

## 2. Methods

This multi-center follow-up observational study investigated inpatients with traumatic fractures. We retrospectively analyzed the medical records of patients with traumatic fractures who received at least 5-day inpatient care at 5 KM hospitals in Gangnam, Bucheon, Daejeon, Haeundae, and Bundang between March 2017 and August 2020. In addition, we conducted a follow-up questionnaire survey on patients who consented to take part in the survey. All 5 KM hospitals included in the study are accredited by the Ministry of Health and Welfare. Integrative Western medicine (WM) and KM treatments are administered in these hospitals based on modern medical diagnostic technologies.

### 2.1. Study population

#### 2.1.1. Inclusion criteria.

Patients aged 19–85 years who had a fracture caused by an injury; received at least a 5-day inpatient care at the Gangnam, Bucheon, Daejeon, Haeundae, or Bundang branch of Jaseng Hospital of Korean Medicine; who understood the objective and contents of the study; and who consented to participate in the survey were included.

#### 2.1.2. Exclusion criteria.

Patients with pathological or stress fractures; with open fractures caused by trauma; with pain caused by diseases other than fractures (including tumors, fibromyalgia, rheumatoid arthritis, and gout); and who could not provide appropriate responses to the questionnaire were excluded. Patients with insufficient data who were determined to be unfit for participation by the researcher were also excluded.

### 2.2. Patient and public involvement

The patients or public were not involved in designing the study, recruitment, or study conduction. Additionally, there are no plans to announce the results of the study via any other platform.

### 2.3. Analysis of medical records

We collected the following medical information from the electronic medical records (EMRs): sex, age, and type of insurance; date of admission, date of discharge, length of hospital stay (in days), and diagnosis; previous medical history; date and cause of the onset of current symptoms; admission to a WM hospital prior to inpatient care; if so, treatment details; details of treatments received during inpatient care at the applicable KM hospital (type and frequency of treatment); numeric rating scale (NRS) score, EuroQol 5-Dimension 5-Level (EQ-5D-5L) score, Oswestry Disability Index (ODI), Neck Disability Index (NDI), Shoulder Pain and Disability Index (SPADI), and Western Ontario and McMaster Universities Arthritis Index (WOMAC) score for the fracture site from admission to discharge.

### 2.4. Treatment

Patients received treatments during their inpatient care according to the integrative KM treatment protocol. Treatment was determined by KM doctors based on patients’ individual conditions. Integrative KM treatments administered during inpatient care included acupuncture, electroacupuncture, pharmacopuncture, cupping, herbal steam therapy, chuna, and herbal medicine. All treatments were recorded in the EMRs, which were used for treatment investigation.

### 2.5. Follow-up questionnaire survey

A follow-up questionnaire survey was conducted after discharge to investigate the status of the fracture site and patients’ satisfaction with KM treatment. The survey was conducted via telephone calls or online using Google forms. The survey contents included recommendation of surgery before admission or after discharge, and if the patient had undergone surgery, the type of surgery; time frame of surgery; any radiological examination after discharge for reevaluation, and if so, radiological findings; treatment within the past 3 months, and if so, the type and frequency; Patient Global Impression of Change (PGIC) score and satisfaction with KM treatment; and current status of fracture site (NRS score, EQ-5D-5L score, ODI, NDI, SPADI, WOMAC, shortened Disabilities of the Arm, Shoulder, and Hand questionnaire [Q-DASH], Foot Function Index [FFI], Hip Disability and Osteoarthritis Outcome Score [HOOS], and items about ribs and sternum).

Among the questions for assessing the fracture site’s status, the NRS and EQ-5D-5L were implemented in all patients regardless of the fracture site, while all other questions were tailored according to each patient’s fracture site.

### 2.6. Primary outcome

NRS is a numeric scale, ranging from 0 to 10, used to rate the severity of pain, with 0 representing “no pain” and 10 representing “the worst possible pain.” The NRS scores of the fracture sites measured every 2 weeks from admission to discharge were obtained from the EMRs and were also measured during the follow-up survey. The effectiveness of integrative KM treatment was assessed based on comparison of the NRS scores between admission and discharge.^[11]^

### 2.7. Secondary outcomes

For the EQ-5D-5L, ODI, NDI, WOMAC, and SPADI, the scores measured every 2 weeks from admission to discharge were obtained from the EMRs, and the scores were additionally measured during the follow-up survey. Where possible, the Korean version, with proven validity and reliability, of each index was used.

The EQ-5D-5L, consisting of 5 items (mobility, self-care, usual activities, pain, and anxiety/depression), is used to assess patients’ QoL. Each item is rated on a 5-point scale. The Korean version was used.^[12,13]^

The ODI, consisting of 10 items, is designed to assess the functional status of patients with low back pain. Each item consists of 6 levels, ranging from 0 to 5 points, with higher scores indicating more severe disabilities. The Korean version was used for patients with lumbar, thoracic, or sacral spine fractures.^[14,15]^

The NDI, consisting of 10 items, is designed to assess neck disability. Each item consists of 6 levels, ranging from 0 to 5 points, with higher scores indicating more severe disabilities. The Korean version was used for patients with cervical spine fractures.^[16,17]^

The WOMAC, consisting of 3 subscales (pain, 5 questions; stiffness, 2 questions; and physical function, 17 questions), is designed to assess knee joint function. Each item is rated on a 5-point scale, with higher scores indicating a poorer knee joint status. The Korean version was used for patients with knee fractures.^[18,19]^

The SPADI, consisting of 2 dimensions (pain, 5 questions; and functional activities, 8 questions), is designed to assess shoulder pain and disability. Each question is rated on a scale of 0 to 10 points, with higher scores indicating more severe shoulder disabilities. The Korean version of the SPADI questionnaire was used for patients with shoulder fractures.^[20,21]^

The Q-DASH is a shortened version of the DASH and is designed to assess the function of patients with upper extremity disorders. It consists of 11 basic questions and 2 optional scales (work-related and sports/arts activities; 4 questions each). Each question is rated on a scale of 0 to 5 points, with higher scores indicating more severe upper extremity disabilities. We used the Korean version. Patients with fractures of the clavicle, arm, elbow, wrist, or hand were evaluated based on the basic questions of the Q-DASH during the follow-up survey.^[22,23]^

The FFI is designed to assess the impact of foot pain on the activities of daily living (ADL). It consists of 3 subscales (pain, disability, and activity limitation) with 9 items each. The Korean version was used and had only 2 validated subscales (pain and disability). Each item was rated on a scale of 0 to 9 points, and the total score for each subscale was converted to a scale of 0 to 100 points, with higher scores indicating poorer foot functions. In this study, only the 9 items in the pain subscale of FFI were used to evaluate patients with fractures of the foot or ankle during the follow-up survey.^[24,25]^

HOOS is designed to assess the extent to which hip problems impact ADL. It includes 10 items on pain, 5 items on symptoms, 17 items on ADL, 4 items on sport and recreation, and 4 items on QoL. Each item consists of 5 levels, from 0 to 4 points, and the total score for each subscale was converted to a scale of 0 to 100 points, with lower scores indicating better status. The Korean version was used to evaluate patients with hip fractures, including fractures of the pelvis, ilium, ischium, or pubic bone, during the follow-up survey.^[26,27]^

The following 4 items were included in the survey based on typical symptoms that appear in cases involving rib or sternal fractures, as reported in a previous study^[28]^: deep breathing, coughing, turning over the body, and sleeping. Each item consisted of 4 levels.

During the follow-up survey, PGIC was used to investigate the patients’ subjective opinions regarding fracture-related pain and functional improvement. It is used for the subjective assessment of the impression of change based on 7 levels.^[29]^

### 2.8. Ethical statement

This study was approved by the Institutional Review Board of Jaseng Hospital of Korean Medicine (approval no.: JASENG 2020-09-016; approval date: January 21, 2021) and was registered at ClinicalTrials.gov (NCT04855058). The EMRs of patients who had given prior consent to use their medical information for research purposes were analyzed. The follow-up questionnaire survey was conducted for discharged patients who consented to survey participation after a full understanding of the use of their personal information. If any patient refused to provide consent for the study, the use of their personal information was discontinued, and such information was destroyed immediately.

### 2.9. Statistical analysis

For all statistical analyses, two-sided tests were performed, with the significance level set at 5%. Continuous variables are presented as means and standard deviations, while categorical variables are presented as frequencies and percentage. The fractures were repeatedly measured from at least one site on each patient, while a linear mixed model that included subject random intercept was used to test the outcomes with respect to a decrease from the baseline. The outcomes (NRS score, EQ-5D-5L score, and other assessment indices) at admission, discharge, and the follow-up survey were expressed as least square estimates.

Moreover, a survival analysis was performed to estimate the mean time until achieving a minimal clinically important difference (MCID) in the NRS. The MCID was considered a 30% decrease relative to the baseline NRS score.^[30–32]^ Discharge was considered censoring, while patients with any missing values after the baseline were considered to show no MCID until discharge.

The length of hospital stay, intervention, and herbal medicine prescription from the EMRs were assessed using a frequency analysis. In the questionnaire survey, all survey items, except the assessment variables, were analyzed using a frequency analysis.

## 3. Results

### 3.1. Study flow

**Figure 1.**
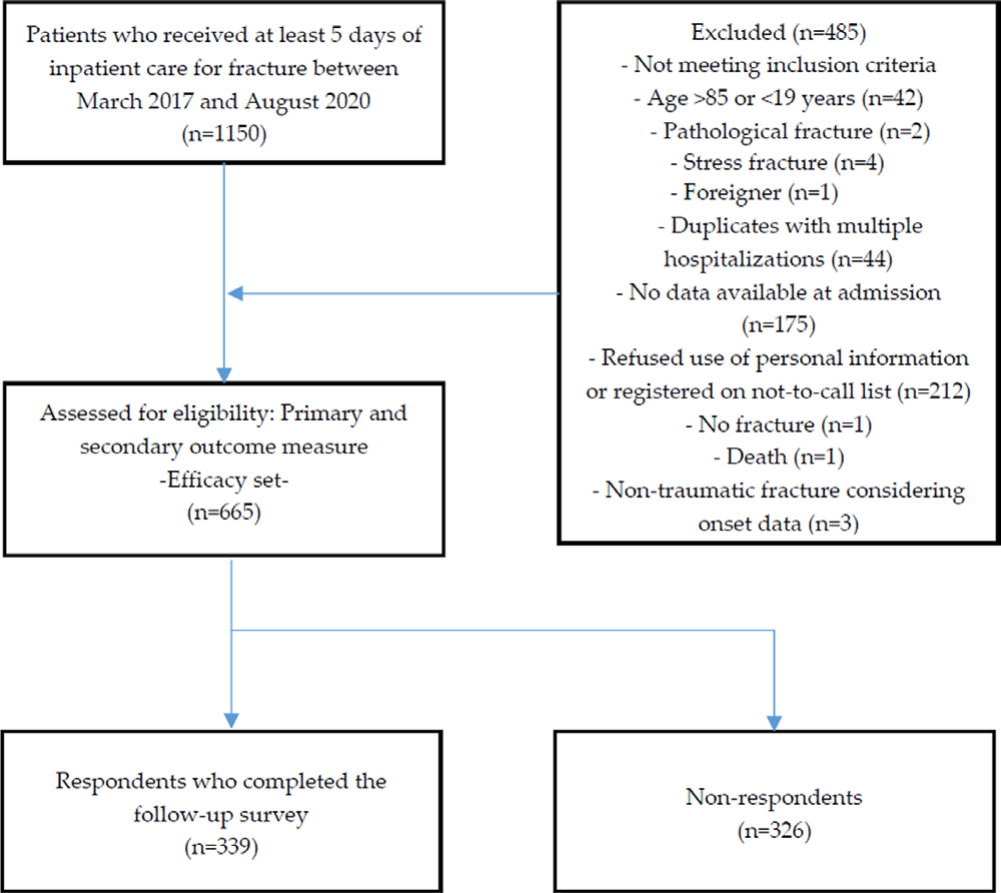
Flow chart of the study.

This study included 1150 patients who received at least 5-day inpatient care for traumatic fractures at one of the 5 KM hospitals included in the study. Patients who did not meet the inclusion and exclusion criteria, those who refused the use of personal information or registered on the do-not-call list, duplicate patients with multiple hospitalizations, and those with no baseline NRS score were excluded. Subsequently, of the 665 included patients, 339 participated in the follow-up survey (shown in Fig. 1).

### 3.2. Baseline characteristics

The study population included 406 women (61.6%), and the mean age was 56.9 ± 16.4 years. Regarding the type of insurance reported on the questionnaire, automobile insurance was the most common response (n = 393, 59.1%), followed by national health insurance (n = 257, 38.6%). The mean duration from onset to hospitalization was 27.8 ± 163.1 days. Overall, 537 patients (80.8%) had visited a WM hospital before admission to a KM hospital, among which 316 patients (47.5%) were diagnosed with fractures at a WM hospital. There were 39 patients (5.9%) who had undergone surgery, with pin fixation (n = 29, 4.4%) being the most common type of surgery. Moreover, 33 (5.0%) and 49 patients (7.4%) were treated with casts and splints, respectively (Table 1).

**Table 1:** Baseline characteristics of patients with traumatic fractures (n = 665).

Characteristics		N (%)
Sex	Female	406 (61.1)
	Male	259 (38.9)
	Mean ± SD	56.91 ± 16.35
Age (yr)	<30	43 (6.5)
	31–40	80 (12.0)
	41–50	93 (14.0)
	51–60	120 (18.0)
	61–70	157 (23.6)
	>70	172 (25.9)
Insurance	Automobile insurance	393 (59.1)
	National health insurance	257 (38.6)
	Medical care insurance	8 (1.2)
	Workers’ compensation insurance	7 (1.1)
	Mean ± SD	27.82 ± 163.06
Duration from onset to admission (days)	>7	337 (50.7)
	8–14	107 (16.1)
	15–31	123 (18.5)
	32–92	57 (8.6)
	93–365	21 (3.2)
	>365	5 (0.8)
	Unknown	15 (2.3)
Previous visit to Western medicine hospital	Yes	537 (80.8)
	No	128 (19.2)
Previous diagnosis of fracture*	Yes	316 (47.5)
	No	349 (52.5)
Previous surgery*	Yes	39 (5.9)
	No	626 (94.1)
Type of surgery*	Pin fixation	29 (4.4)
	Cement augmentation	2 (0.3)
	Others	1 (0.2)
	Unknown	7 (1.1)
Previous cast or splint application*	Cast	33 (5.0)
	Splint	49 (7.4)
	None	583 (87.7)

SD = standard deviation.

* Diagnosis and treatment at a Western medicine hospital visited before admission to a Korean medicine hospital.

### 3.3. Post-treatment value changes

The mean NRS score for all fracture sites significantly reduced by 2.05 points (95% confidence interval [CI]: 1.94–2.17; *P* < .001) and 4.41 points (95% CI: 4.24–4.57; *P* < .001), at discharge and follow-up, respectively, from that at admission. A subgroup analysis was performed for fracture sites with sufficient sample sizes: the ribs/sternum, lumbar spine, thoracic spine, ankle/foot, wrist/hand, and knee. The results showed a significant post-treatment reduction in NRS scores at all 6 fracture sites (*P* < .001).

The mean ODI significantly decreased by 15.08 points (95% CI: 13.10–17.07; *P* < .001) and 39.26 points (95% CI: 36.30–42.21; *P* < .001) at discharge and follow-up, respectively, from that at admission. The mean WOMAC significantly decreased by 8.69 points (95% CI: 0.42–16.95; *P* = .04) and 42.23 points (95% CI: 29.52–54.94; *P* < .001) at discharge and follow-up, respectively, from that at admission. The mean EQ-5D-5L score significantly increased by 0.12 points (95% CI: 0.01–0.23; *P* = .038) and 0.18 points (95% CI: 0.03–0.33; *P* = .022) at discharge and follow-up, respectively, from that at admission (Table 2).

**Table 2:** Post-treatment changes in pain, functional impairment, and QoL.

		Time	N*	Value	Difference	*P* value
NRS	Total	Admission	764	6.01 (5.91–6.10)	–	–
		Discharge	764	3.95 (3.83–4.07)	2.05 (1.94–2.17)	<.001
		Follow-up	391	1.60 (1.43–1.76)	4.41 (4.24–4.57)	<.001
	Rib, sternum	Admission	255	5.93 (5.77–6.09)	–	–
		Discharge	255	4.09 (3.87–4.30)	1.84 (1.62–2.06)	<.001
		Follow-up	142	1.28 (0.99–1.57)	4.65 (4.35–4.94)	<.001
	Lumbar spine	Admission	214	6.14 (5.96–6.32)	–	–
		Discharge	214	3.69 (3.49–3.89)	2.45 (2.25–2.65)	<.001
		Follow-up	98	1.59 (1.30–1.89)	4.55 (4.26–4.84)	<.001
	Thoracic spine	Admission	89	6.07 (5.78–6.36)	–	–
		Discharge	89	3.75 (3.42–4.08)	2.32 (1.99–2.64)	<.001
		Follow-up	35	2.15 (1.62–2.67)	3.92 (3.39–4.45)	<.001
	Ankle, foot	Admission	65	6.06 (5.71–6.41)	–	–
		Discharge	65	4.33 (3.94–4.72)	1.73 (1.34–2.13)	<.001
		Follow-up	30	1.49 (0.92–2.06)	4.57 (4.01–5.14)	<.001
	Wrist, hand	Admission	37	5.70 (5.30–6.11)	–	–
		Discharge	37	4.30 (3.71–4.88)	1.41 (0.82–2.00)	<.001
		Follow-up	26	1.67 (0.96–2.38)	4.04 (3.32–4.75)	<.001
	Knee	Admission	29	6.04 (5.63–6.44)	–	–
		Discharge	29	4.07 (3.52–4.62)	1.97 (1.42–2.51)	<.001
		Follow-up	12	1.95 (1.07–2.84)	4.08 (3.20–4.97)	<.001
ODI†		Admission	275	54.43 (52.25–56.61)	–	–
		Discharge	275	39.35 (37.36–41.33)	15.08 (13.10–17.07)	<.001
		Follow-up	125	15.17 (12.21–18.13)	39.26 (36.30–42.21)	<.001
WOMAC‡		Admission	21	58.27 (49.77–66.78)		
		Discharge	21	49.59 (41.33–57.85)	8.69 (0.42–16.95)	.04
		Follow-up	9	16.04 (3.33–28.76)	42.23 (29.52–54.94)	<.001
EQ-5D-5L§		Admission	603	0.72 (0.46–0.99)		
		Discharge	603	0.84 (0.73–0.95)	0.12 (0.01–0.23)	.038
		Follow-up	317	0.90 (0.75–1.05)	0.18 (0.03–0.33)	.022

All values are presented as means and 95% CIs. Differences are expressed as mean changes (95% CIs) compared with the admission baseline values.

CI = confidence interval, EQ-5D-5L = EuroQol 5-dimension 5-level, NRS = Numeric rating scale, ODI = Oswestry disability index, QoL = Quality of Life, SD = standard deviation, WOMAC = Western Ontario and McMaster Universities Arthritis Index.

* Number of fracture episodes counted based on fracture diagnosis.

† Investigation of patients with fractures of the lumbar spine, thoracic spine, or sacrum.

‡ Investigation of patients with knee fractures.

§ Investigation of all patients with fractures.

The NRS score, NDI, and SPADI for fractures of the sacrum, cervical spine, shoulder, clavicle, arm, and pelvis could not be statistically analyzed owing to the small number of cases, but the scores showed an overall decreasing trend (Table S1, Supplemental Digital Content, http://links.lww.com/MD/J408, which illustrates changes in outcomes at admission, discharge, and follow-up). For fractures of the rib (n = 115) and sternum (n = 26) investigated during the follow-up survey, assessment of symptoms for 4 items (deep breathing, coughing, turning over the body, and sleeping) showed responses in the order of “not interfered at all” and “slightly interfered” (Table S2, Supplemental Digital Content, http://links.lww.com/MD/J409, which illustrates level of symptoms in patients with fractures of the rib and sternum). Regarding the scores obtained during the follow-up survey, the Q-DASH (n = 42) score was 10.12 ± 15.48 points, and the FFI (n = 23) was 12.65 ± 14.65 points. The scores for each subscale of the HOOS (n = 6) were 78.3 ± 22.1 for pain, 79.2 ± 19.6 for symptoms, 82.4 ± 20.5 for ADL, 77.1 ± 22.9 for sport and recreation, and 70.8 ± 23.6 for QoL.

### 3.4. Survival analysis

**Figure 2.**
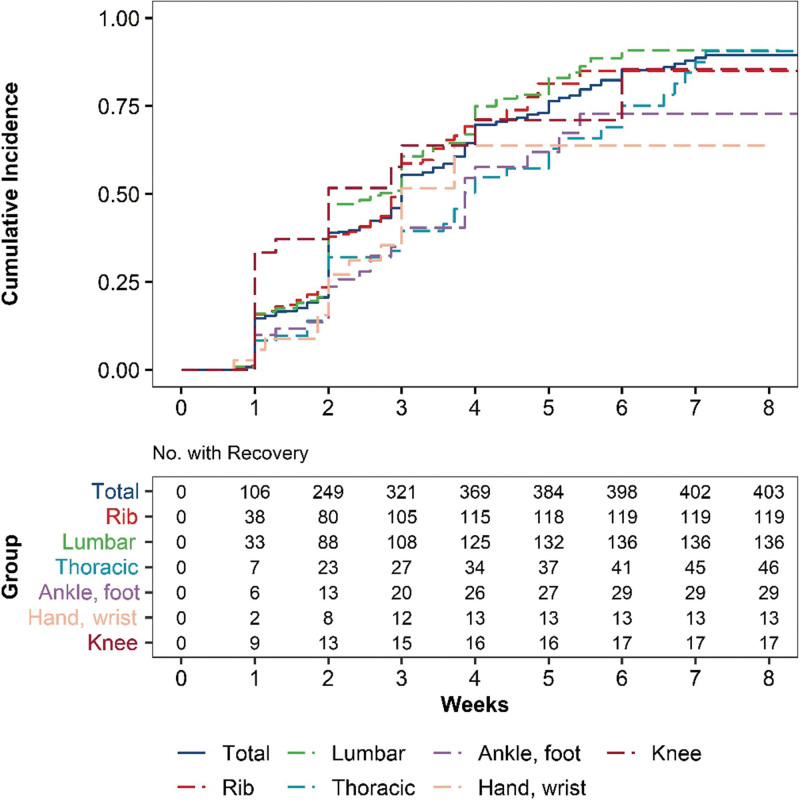
Survival curves. Recovery was defined as a reduction of more than 30% in the NRS scores. NRS = numeric rating scale.

The MCID was set as a 30% decrease in the NRS score, and survival analysis was performed to measure the time until achieving the MCID. The analysis was performed on 6 fracture sites: the rib/sternum, lumbar spine, thoracic spine, ankle/foot, hand/wrist, and knee. The median time to achieve the MCID relative to the mean NRS score for all fracture sites was 21 days (95% CI: 20–21). The median time for the knee was 14 days (95% CI: 9–NA), which was the fastest, followed in order by 19 days (95% CI: 14–21) for the lumbar spine, 21 days (95% CI: 19–23) for the rib/sternum, 21 days (95% CI: 19–NA) for the hand/wrist, 27 days (95% CI: 21–40) for the thoracic spine, and 27 days (95% CI: 21–NA) for the ankle/foot (shown in Fig. 2; Table 3).

**Table 3:** Median time to MCID achieved by patients with traumatic fractures.

	N*	Case† (%)	Median time‡ (days, 95% CI)
Total	764	406 (53.14)	21 (20–21)
Knee	29	17 (58.62)	14 (9–NA)
Lumbar spine	214	136 (63.55)	19 (14–21)
Rib, sternum	255	119 (46.67)	21 (19–23)
Wrist, hand	37	13 (35.14)	21 (19–NA)
Thoracic spine	89	48 (53.93)	27 (21–40)
Ankle, foot	65	30 (46.15)	27 (21–NA)

The MCID criterion is a reduction in the NRS score by 30%.

MCID = minimal clinically important difference, NA = not applicable, NRS = numeric rating scale.

* Number of fracture episodes counted based on fracture diagnosis.

† Number of cases in which MCID in NRS was achieved.

‡ Time required for half of the cases to achieve MCID.

### 3.5. Treatments

The mean length of hospital stay was 22.5 ± 13.9 days (median: 20 days; Table S3, Supplemental Digital Content, http://links.lww.com/MD/J410, which illustrates length of hospital stay by body part), and the patients received integrative KM treatment, including acupuncture, pharmacopuncture, and herbal medicine, during their inpatient care. The most frequently prescribed interventions were acupuncture, cupping, electroacupuncture, and pharmacopuncture (Table S4, Supplemental Digital Content, http://links.lww.com/MD/J411, which illustrates Interventions administered during the hospital stay). The most frequently prescribed herbal medicines were Ansinjitong-tang, Jeopgol capsules, and Gwanjeol-go (Table S5, Supplemental Digital Content, http://links.lww.com/MD/J412, which illustrates 10 high-frequency herbal medicine prescriptions).

### 3.6. Follow-up survey

The median number of days from discharge to the follow-up survey was 784 days, with 339 patients responding. Because the respondents included patients with multiple fractures, the number of responses for fracture sites was 370. The details of surgery and radiological examination were investigated for each fracture site, while treatment within 3 months and each patient’s PGIC scores were investigated.

The number of cases with surgery recommended before admission was 46/370 (12.43%), and of these, surgery was performed before admission in 30 cases (8.11%). The most commonly performed surgery was pin fixation (n = 21, 5.68%), followed by cement augmentation (n = 6, 1.62%). The number of cases recommended for surgery after discharge was 11/370 cases (2.97%). The most commonly performed surgery was cement augmentation (n = 8, 2.16%), followed by pin fixation (n = 1, 0.27%).

Additional radiological examination after discharge for progress assessment was performed in 163 cases (44.05%), of which findings showed complete recovery in 96 cases (25.95%), recovery with good progress in 56 cases (15.14%), and the need for additional treatment or surgery in 10 cases (2.70%). The number of patients who received treatment within 3 months from the survey was 53/339 (15.63%), and the mean number of treatments received in 3 months was 18.88 ± 10.65. The most frequently prescribed interventions were acupuncture (n = 33, 9.73%), physical therapy (n = 30, 8.85%), and cupping (n = 20, 5.90%).

For PGIC after KM inpatient care, the responses of 307 patients were “minimally improved” or better, accounting for 90.56% of all responses (Table 4).

**Table 4:** Follow-up survey.

Period from discharge to follow-up (n = 339)		Days
Maximum		1582
Median		784
Minimum		211
**Surgery (n = 370**)*		**N (%**)
Recommended surgery before admission		46 (12.43)
Underwent surgery before admission		30 (8.11)
Type of surgery†	Pin fixation	21 (5.68)
	Cement augmentation	6 (1.62)
	Others	2 (0.54)
	Unknown	2 (0.54)
Recommended surgery after discharge		13 (3.51)
Underwent surgery after discharge		11 (2.97)
Type of surgery†	Cement augmentation	8 (2.16)
	Pin fixation	1 (0.27)
	Others	2 (0.54)
	Unknown	0 (0)
**Imaging examination after discharge (n = 370**)*		**N (%**)
Yes		163 (44.05)
No		207 (55.95)
Examination results		
Recovered completely		96 (25.95)
Recovering well according to the progress		56 (15.14)
Additional treatment or surgery is needed		10 (2.70)
Others		1 (0.27)
**Patients received treatment in recent 3 months (n = 339**)		**N (%**)
Yes		53 (15.63)
No		286 (84.37)
**Number of treatments (mean ± SD**)		**18.88 ± 10.65**
Type of treatment†		
Acupuncture		33 (9.73)
Physical therapy		30 (8.85)
Cupping		20 (5.90)
Analgesic-per oral		12 (3.54)
Pharmacopuncture		11 (3.24)
Herbal medicine		10 (2.95)
Chuna manual therapy		6 (1.77)
Injection therapy		5 (1.47)
Others		5 (1.47)
Moxa		4 (1.18)
**Patients’ Global Impression of Change (n = 339**)		**N (%**)
Very much improved		104 (30.68)
Much improved		142 (41.89)
Minimally improved		61 (17.99)
No change		29 (8.55)
Minimally worse		0 (0)
Much worse		0 (0)
Very much worse		0 (0)
**Unknown**		**3 (0.88**)

SD = standard deviation.

* Number of responses to the survey for each fracture site.

† Multiple answers allowed.

## 4. Discussion

In this study, assessment of various indicators of pain, functional impairment, and QoL showed improvement in patients with traumatic fractures who received integrative KM treatment. Moreover, analysis of the long-term therapeutic effect through a follow-up survey showed that the patients exhibited relatively good post-discharge progress.

In the post-treatment analysis, the results showed decreased NRS, ODI, WOMAC, and EQ-5D-5L scores at discharge and during the follow-up survey, compared with the scores at admission. The Q-DASH scores, HOOSs, and FFI, which were investigated for outcomes in each site during the follow-up survey, could not be compared and analyzed, as they were not measured at discharge. However, considering that a study on patients with humeral shaft fractures viewed Q-DASH scores < 15 as recovery,^[33]^ Q-DASH scores of 10.12 in this study can be viewed as indicating a relatively recovered state.

In this study, MCID was set as a 30% decrease in NRS scores, and the time required to achieve MCID or better was measured. Considering that the median survival time for all fractures was 21 days (95% CI: 20–21) and the mean length of hospital stay was 22.5 ± 13.9 days, it was determined that half of the patients showed improvement with a ≥ 30% decrease in NRS scores before discharge. In the analysis of inpatient care, the length of hospital stay was the longest with fractures of the thoracic spine, followed by the pelvis, lumbar spine, and sacrum, while fractures of the trunk tended to require longer hospital stays than fractures of the limbs.

Per the follow-up survey, 46 cases (12.43%) were recommended surgery before admission, and among these, 30 cases underwent surgery (8.11%). Meanwhile, 13 cases (3.51%) were recommended surgery after discharge, and among these, 11 actually underwent surgery (2.97%). Among 16 cases (4.32%) in which the patients were recommended surgery before admission but did not undergo surgery, patients did not receive a recommendation for surgery after discharge in 13 cases (3.51%). Among the 3 cases which were recommended surgery after discharge, only 2 (0.54%) underwent surgery. The number of patients who had received treatment within 3 months of the follow-up survey was 53 (15.63%), while 286 patients (84.37%) did not receive treatment during that period. Regarding PGIC items, 307 (90.56%) patients responded “minimally improved” or better after integrative KM treatment. Based on such findings, it was determined that the conditions of most of the patients were maintained to the point of not requiring any additional treatment when the follow-up survey was conducted.

The fracture healing process typically comprises 3 phases: the inflammatory, reparative, and remodeling phases. The inflammatory phase lasts for ~7 days after the fracture, during which time, hematomas and inflammatory exudates are formed from the ruptured blood vessels. Inflammatory cells and fibroblasts are mobilized, and hematoma at the fracture site is gradually replaced by granulation tissues, while osteoclasts replace bone tissue at the margins of bone fragments. The reparative phase is also referred to as the callus phase. During the soft callus phase, ~3 weeks after the fracture, pain and swelling subside and soft calluses form, while infiltration of blood flow and capillaries into the calluses and the number of cells increase. Subsequently, during the hard callus phase, the soft calluses are converted into hard calcified tissue through endochondral and intramembranous ossification. The remodeling phase starts after hard union of the fracture and continues for several months to years until the bone returns to its original form.^[1,34–36]^

The KM treatment approach aids in reducing pain and promoting recovery during the healing process. Herbal medicine is considered to be helpful in fracture healing processes. Previous experimental studies on herbal medicine showed that callus formation occurred faster in the herbal medicine group than in the control group.^[37,38]^ Measurement of radical scavenging activity of herbal extracts showed a dose-dependent increase in antioxidant activity and a significant decrease in the production of the inflammatory mediator interleukin-1β, indicating that herbal extracts can help relieve inflammatory responses accompanying fractures.^[39,40]^ Osteogenesis during the reparative phase can be facilitated by bone morphogenetic protein type 2 that mediates the differentiation of osteoblasts and activates transforming growth factor-β1 and Sox9 that regulates the transcription of type 2 collagen. Moreover, bone union during the reparative phase and maintenance of stability during the remodeling phase can be increased through the collagen type II alpha 1 chain that regulates endochondral ossification^[37,41]^ and expression of osteocalcin that contributes to bone remodeling and the change to hard calluses.^[41,42]^

According to a study that used the Taiwanese National Health Insurance Research Database, the fracture patient group that received herbal medicine treatment had lower inpatient care costs within 6 months from the accidents than the control group.^[43]^ A study on only patients with hip fractures showed that taking herbal medicine reduced the risk of mortality, rehospitalization, and re-operation.^[44]^ In an RCT including 170 patients with rib fractures, the treatment time and cost until the visual analog scale (VAS) score was reduced by half were lower in the herbal medicine group than in the nonsteroidal anti-inflammatory drugs group.^[45]^ In this study, the most frequently prescribed herbal medicines were Ansinjitong-tang (n = 246, 36.99%), Jeopgol capsules (n = 132, 19.85%), and Gwanjeol-go (n = 105, 15.79%). Ansinjitong-tang is effective in treating distal radius fractures,^[43]^ while Jeopgol capsules and Gwanjeol-go are effective in treating lumbar burst fractures.^[44]^

Several studies have been published on the effects of acupuncture for various fracture sites. A study that analyzed Taiwanese National Health Insurance Research Database for the effects of acupuncture on patients with hip fractures showed that the patient group that received 6 sessions of acupuncture treatment after fractures showed significantly lower mortality, rehospitalization, and re-operation rates than its counterparts.^[46]^ An RCT on the efficacy of acupuncture for intertrochanteric fracture revealed that the group that received a combination of acupuncture and aspirin showed significantly lower VAS scores at postoperative days 5 and 7 and significantly higher Harris scores and modified Barthel indexes 2 months postoperatively, than the control group that received only aspirin.^[9]^ A systematic review of patients with humeral fractures indicated that the acupuncture group showed significantly lower pain indicator scores than the control groups using conventional drugs such as analgesics, physical therapy, or placebos.^[10]^ Such findings suggested that acupuncture alone or in combination with conventional treatment can help in pain alleviation and recovery of function and daily living ability. The mechanism by which acupuncture helps with recovery from fractures has been reported to involve inhibition of free oxygen radicals and regulation of antioxidant enzymes^[47]^ and reduction in the levels of inflammatory factors, such as C-reactive protein and tumor necrosis factor-α.^[9]^

Most studies on pharmacopuncture for fractures used pharmacopuncture combined with other treatment interventions. Consequently, it is difficult to analyze the sole effects of pharmacopuncture. In an animal study using an intervention combining pharmacopuncture and herbal medicine, the group that received both pharmacopuncture and herbal medicine showed a significant increase in serum alkaline phosphatase levels compared with the control group that did not receive any intervention. In addition, increased osteogenesis and higher transforming growth factor-β1 expression in the fracture site were found in histological observations.^[48,49]^ In a retrospective review of patients with thoracic or lumbar compression fractures, the group that received complex pharmacopuncture therapy showed a significant decrease in VAS scores and a significant increase in ODI, compared with the control group that received only KM treatment.^[50]^

The present study population included 665 patients, but only 50.98% (n = 339) took part in the follow-up survey. There is a limitation in that the effects by gender or age could not be compared due to the different fracture sites and low follow-up rates. The median time from discharge to the follow-up survey was 784 days, while the maximum was 1582 days. Therefore, the possibility of recall bias for surgery and treatment after discharge cannot be dismissed. Moreover, because the records regarding inpatient care were retrospectively reviewed, it was difficult to obtain data regarding the type of fracture or displacement. Among the outcome tests, surveys using questionnaires such as Q-DASH, FFI, and HOOS were not performed during hospitalization, and as a result, comparative analysis with pretreatment data was impossible. Due to this, there are limitations to reporting the effect primarily on the NRS and not fracture-specific questionnaires or area-function-specific questionnaires. In addition, it was analyzed by setting the MCID of NRS to 30%, but this is a setting based on chronic pain, and it was difficult to set an MCID that was suitable for fracture. Other limitations of this study included the fact that the study was not conducted in a controlled environment and that there was no control group as a comparator. The integrative KM treatment applied in this study included several different interventions that were applied together, and as a result, it was difficult to distinguish the therapeutic effects of each intervention. It was difficult to clearly distinguish the natural process of fracture healing from the additional effects of treatment. Furthermore, our inability to completely exclude the effects of other treatments received during the follow-up was a limitation of this study, even though treatment history within 3 months of filling the questionnaire was investigated.

Under Korea’s dual healthcare system, patients with fractures often choose KM treatment as they await natural bone union, if they do not need surgery or to improve pain and function in the fracture site postoperatively. Among all patients with fractures, the proportion of those who receive treatment at KM institutions has gradually increased, from 1.02% in 2016 to 1.38% in 2020; however, the percentage is still low.^[4]^ Moreover, studies on KM treatment for fractures are also lacking. Through this study, the treatment modes for fractures administered in KM hospitals, such as fracture sites and duration of treatment, were identified, and the sustained effects of KM treatment were identified through a follow-up survey. Furthermore, various assessment indicators, including the EQ-5D-5L scores, questionnaires for different fracture sites, and questionnaires on satisfaction, were used in addition to the NRS for multi-dimensional assessment of the effectiveness of integrative KM treatment. Therefore, the study findings could be used as reference data for further studies. To establish evidence for the effectiveness and safety of KM treatment for fractures, registry studies or RCTs using patients undergoing other surgical techniques or conservative therapy (including the use of analgesics) as control groups are needed.

In conclusion, integrative KM treatment as a conservative treatment might improve the pain, function, and QoL in patients with traumatic fractures, and its sustained long-term effects were identified through a follow-up survey. Although further research with a high level of evidence is warranted, integrative KM treatment can be considered as a conservative treatment modality for the management of traumatic fractures.

## Author contributions

**Conceptualization:** Yoon Jae Lee, Jinho Lee.

**Formal analysis:** Eun-San Kim.

**Methodology:** Eun-San Kim.

**Supervision:** Jinho Lee, In-Hyuk Ha.

**Writing – original draft:** Min Kyung Kim, Kyoung Sun Park.

**Writing – review & editing:** Gyu Cheol Choi, Jae Eun Yu, Hee Won Lee, Yong Su Kwon, Hyo Seung Huh, Suna Kim.

## Supplementary Material
