# Supplementary material for: Long-term follow-up of inpatients with traumatic fractures who received integrative Korean Medicine treatment: A retrospective analysis and questionnaire survey study
Source: Medicine (Baltimore). 2023 Oct 13;102(41):e34530. doi: 10.1097/MD.0000000000034530 (PMC10578701; doi:10.1097/MD.0000000000034530)
Supplement: Supplementary file 1 [file medi-102-e34530-s001.pdf]

Table S1: Changes in outcomes at admission, discharge, and follow-up

		Time	N*	Mean ± SD
NRS	Sacrum	Admission	9	6.56 ± 1.33
		Discharge	9	4.11 ± 1.76
		Follow-up	5	2.20 ± 2.28
	Cervical spine	Admission	12	5.92 ± 1.24
		Discharge	12	3.58 ± 1.24
		Follow-up	7	2.43 ± 2.15
	Shoulder	Admission	10	6.30 ± 1.34
		Discharge	10	4.80 ± 1.40
		Follow-up	6	2.00 ± 2.45
	Clavicle	Admission	12	5.67 ± 1.72
		Discharge	12	3.58 ± 1.08
		Follow-up	8	3.25 ± 2.25
	Arm	Admission	13	5.54 ± 1.05
		Discharge	13	3.92 ± 1.93
		Follow-up	11	1.73 ± 1.68
	Pelvis	Admission	13	6.00 ± 1.00
		Discharge	13	3.38 ± 1.04
		Follow-up	8	1.12 ± 1.36
NDI†		Admission	11	48.53 ± 8.98
		Discharge	11	39.28 ± 16.35
		Follow-up	6	33.33 ± 10.33
SPADI‡		Admission	8	68.85 ± 25.56
		Discharge	7	58.33 ± 24.86
		**Follow-up**	**5**	**22.62 ± 23.88**

** Number of fracture episodes counted based on fracture diagnosis*

† Investigation of patients with fractures of the cervical spine

‡ Investigation of patients with shoulder fractures

NRS: Numeric rating scale; NDI: Neck disability index; SPADI: Shoulder pain and disability index; SD: Standard deviation
