# Supplementary material for: Long-term follow-up of inpatients with traumatic fractures who received integrative Korean Medicine treatment: A retrospective analysis and questionnaire survey study
Source: Medicine (Baltimore). 2023 Oct 13;102(41):e34530. doi: 10.1097/MD.0000000000034530 (PMC10578701; doi:10.1097/MD.0000000000034530)
Supplement: Supplementary file 2 [file medi-102-e34530-s002.pdf]

Table S2: Level of symptoms in patients with fractures of the rib and sternum (n = 136)*

		Not interfered at all	Slightly interfered	Moderately interfered	Severely interfered	Total
Deep breathing	Rib	101 (87.83)	13 (11.30)	0 (0)	1 (0.87)	115 (100)
	Sternum	21 (80.77)	5 (19.23)	0 (0)	0 (0)	26 (100)
Coughing	Rib	101 (87.83)	13 (11.30)	0 (0)	1 (0.87)	115 (100)
	Sternum	20 (76.92)	6 (23.08)	0 (0)	0 (0)	26 (100)
Turning over the body	Rib	98 (85.22)	15 (13.04)	1 (0.87)	1 (0.87)	115 (100)
	Sternum	18 (69.23)	8 (30.77)	0 (0)	0 (0)	26 (100)
Sleeping	Rib	101 (87.83)	12 (10.43)	2 (1.74)	0 (0)	115 (100)
	**Sternum**	**18 (69.23)**	**8 (30.77)**	**0 (0)**	**0 (0)**	**26 (100)**

** 115 cases of rib fractures and 26 cases of sternal fractures, of which, 6 involved fractures of both the ribs and sternum; the total number of responses for rib/sternum fracture was 136. Values are presented as N (%), where N represents the number of fracture episodes based on fracture diagnosis.*
