# Supplementary material for: Long-term follow-up of inpatients with traumatic fractures who received integrative Korean Medicine treatment: A retrospective analysis and questionnaire survey study
Source: Medicine (Baltimore). 2023 Oct 13;102(41):e34530. doi: 10.1097/MD.0000000000034530 (PMC10578701; doi:10.1097/MD.0000000000034530)
Supplement: Supplementary file 3 [file medi-102-e34530-s003.pdf]

Table S3: Length of hospital stay

Length of hospital stay (days)	N*	Median [IQR]
**Total**	**764**	**20.0 [13.0 – 28.0]**
Shoulder	10	13.5 [11.2 – 22.0]
Clavicle	12	16.5 [14.8 – 22.5]
Rib, sternum	255	17.0 [12.0 – 24.0]
Knee	29	17.0 [13.0 – 27.0]
Wrist, hand	37	17.0 [14.0 – 21.0]
Ankle, foot	65	19.0 [12.0 – 31.0]
Others†	6	19.0 [14.8 – 26.2]
Arm	13	20.0 [13.0 – 24.0]
Cervical spine	12	20.5 [13.8 – 24.5]
Thoracic spine	89	21.0 [13.0 – 36.0]
Pelvis	13	23.0 [12.0 – 38.0]
Lumbar spine	214	24.0 [15.0 – 34.8]
**Sacrum**	**9**	**28.0 [22.0 – 29.0]**

* *Number of fracture episodes counted based on fracture diagnosis*

† *Includes craniofacial and coccyx fractures*

IQR: interquartile range
