# Supplementary material for: Long-term follow-up of inpatients with traumatic fractures who received integrative Korean Medicine treatment: A retrospective analysis and questionnaire survey study
Source: Medicine (Baltimore). 2023 Oct 13;102(41):e34530. doi: 10.1097/MD.0000000000034530 (PMC10578701; doi:10.1097/MD.0000000000034530)
Supplement: Supplementary file 4 [file medi-102-e34530-s004.pdf]

Table S4: Interventions administered during the hospital stay (n = 665)

Intervention*	Number of patients [N (%)]	Mean ± SD
Acupuncture	664 (99.85)	20.98 ± 13.48
Cupping	663 (99.70)	23.21 ± 15.72
Electroacupuncture	661 (99.40)	20.67 ± 13.09
Pharmacopuncture	653 (98.20)	19.99 ± 22.78
Herbal medicine†	631 (94.89)	31.00 ± 31.40
Moxa	484 (72.78)	13.03 ± 8.74
Chuna manual therapy	364 (54.74)	12.86 ± 8.57
Physiotherapy	165 (24.81)	10.78 ± 9.33
Herbal steam therapy	112 (16.84)	14.51 ± 11.78
Analgesic-injection	159 (23.91)	—
**Analgesic-per oral**	**182 (27.37)**	**—**

* *Multiple answers allowed*

† Herbal medicine is represented as the number of intervention days

SD: Standard deviation
