# Supplementary material for: Long-term follow-up of inpatients with traumatic fractures who received integrative Korean Medicine treatment: A retrospective analysis and questionnaire survey study
Source: Medicine (Baltimore). 2023 Oct 13;102(41):e34530. doi: 10.1097/MD.0000000000034530 (PMC10578701; doi:10.1097/MD.0000000000034530)
Supplement: Supplementary file 5 [file medi-102-e34530-s005.pdf]

Table S5: Ten high-frequency herbal medicine prescriptions (n = 665)

Name of herbal medicine*	Number of patients [N (%)]	Mean ± SD
Ansinjitong-tang (安神止痛湯)	246 (36.99)	11.52 ± 7.43
Jeopgol-capsule (接骨-capsule)	132 (19.85)	19.93 ± 13.40
Gwanjeol-go (關節膏)	105 (15.79)	20.06 ± 14.56
Whalakhyoryoung-Dan (活絡效靈丹)	96 (14.40)	2.09 ± 3.26
Cheongpajeonsin-bang (靑波煎新方)	95 (14.29)	17.63 ± 14.39
Cheongsinbaro-hwan (靑新 baro 丸)	87 (13.08)	18.36 ± 15.16
Dangguisu-san (當歸鬚散)	75 (11.28)	13.27 ± 8.67
Dangguihwajo-um (當歸和調飮)	68 (10.23)	14.47 ± 9.49
Hwalhyuljungtong-tang (活血止痛湯)	68 (10.23)	12.03 ± 6.21
**Cheongpajeon -H (靑波煎-H)**	**67 (10.08)**	**16.21 ± 14.23**

** Multiple answers allowed. Herbal medicine is represented as the number of intervention days*

SD: Standard deviation
